# Supplementary material for: Hair follicle dermal condensation forms via Fgf20 primed cell cycle exit, cell motility, and aggregation
Source: eLife. 2018 Jul 31;7:e36468. doi: 10.7554/eLife.36468 (PMC6107334; doi:10.7554/eLife.36468)
Supplement: Supplementary file 1. [file elife-36468-supp1.docx]

S2 Table. PrimePCR Probe (Bio-Rad) combinations used in Rt-qPCR analysis

| **Probe combinations** | **Gene** | **Unique Assay ID** |
| --- | --- | --- |
| Combination-1 | *Hprt** | qMmuCEP0054164 |
|  | *Dusp6* | qMmuCIP0029423 |
| Combination-2 | *Eef1** | qMmuCEP0057829 |
|  | *Spred1* | qMmuCEP0055028 |
| Combination-3 | *Gapdh** | qMmuCEP0039581 |
|  | *Etv5* | qMmuCIP0034710 |
|  | *Spry4* | qMmuCEP0054507 |

* reference genes used for calculation of ∆CT values
